# High‐throughput determination of oxygen dissociation curves in a microplate reader—A novel, quantitative approach

**DOI:** 10.14814/phy2.14995

**Published:** 2021-08-24

**Authors:** Simon Woyke, Mathias Ströhle, Hermann Brugger, Giacomo Strapazzon, Hannes Gatterer, Norbert Mair, Thomas Haller

**Affiliations:** ^1^ Department of Anaesthesiology and Critical Care Medicine Medical University of Innsbruck Innsbruck Austria; ^2^ Institute of Mountain Emergency Medicine Eurac Research Bolzano Italy; ^3^ Department of Physiology and Medical Physics Institute of Physiology Medical University of Innsbruck Innsbruck Austria

**Keywords:** blood, hemoglobin, method, oxygen affinity, P50

## Abstract

In vitro determination of the hemoglobin oxygen dissociation curve (ODC) requires highly elaborate, specialized, and costly technical equipment. In addition, there is a lack of methods that combine reliable ODC recordings with high throughput in small blood samples for routine analysis. We here introduce a modified, commercial 96‐well plate with an integrated unidirectional gas flow system specifically adapted for use in fluorescence microplate readers. Up to 92 samples of whole or hemolyzed, buffered or unbuffered blood, including appropriate controls or internal standard hemoglobin solutions, can be analyzed within ~25 min. Oxygen saturation is measured in each well with dual wavelength spectroscopy, and oxygen partial pressure using fluorescence lifetime of commercial oxygen sensors at the in‐ and outlet ports of the gas‐flow system. Precision and accuracy of this method have been determined and were compared with those of a standard method. We further present two applications that exemplarily highlight the usefulness and impact of this novel approach for clinical diagnostics or basic research.

## Graphical abstract

Hemoglobin oxygen dissociation curves are measured using a modified, commercial 96‐well plate with an integrated unidirectional gas‐flow system specifically adapted for use in fluorescence microplate readers. Up to 92 samples of whole or hemolyzed, buffered or unbuffered blood, including appropriate controls or internal standard hemoglobin solutions, can be analyzed within ~25 min.

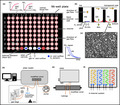

## 1 INTRODUCTION

The oxygen dissociation curve (ODC) describes the reversible binding of, at the maximum, four molecules of oxygen (O_2_) to the tetrameric molecule of hemoglobin (Hb) (Barcroft & Camis, 1909; Imai, 1982; Mairbäurl & Weber, 2012; Severinghaus, 1979). While O_2_ equilibrium curve is the most precise term for the binding characteristics of O_2_ to Hb, ODC, and O_2_ association curve are also used as descriptive terms of the different measurement procedures (Imai, 1982). The most important parameters for characterizing the nonlinear, sigmoidal shape of the ODC are the P50 value (= O_2_ partial pressure, *P*O_2_, at which 50% of Hb is saturated with O_2_) and the Hill coefficient (= maximum slope of the ODC in the Hill plot, a parameter for the cooperativity of O_2_ binding to Hb). Various intrinsic and extrinsic factors have effects on the ODC. Temperature, pH, 2,3‐bisphosphoglycerate (2,3‐BPG), and partial pressure of carbon dioxide (*P*CO_2_) are the four parameters that severely affect O_2_ binding and thus the shape of the ODC (Imai, 1982; Mairbäurl & Weber, 2012). A shift of the curve to the right indicates a decrease in the binding affinity to O_2_, whereas a left shift indicates an increase in O_2_ affinity.

ODC determination is performed in vitro by exposing blood to different gas mixtures, or chemically by adding a substance that eliminates free O_2_ from the sample. In the 1970s and 1980s, scientific data from instruments for ODC measurement were published, almost all of them used absorption measurements for the determination of O_2_ saturation (*S*O_2_) in conjunction either with gas mixtures of known O_2_ content or with actual *P*O_2_ measurements using Clark‐type O_2_ electrodes (Callaghan et al., 1961; Duvelleroy et al., 1970; Lawson et al., 1965; Nelson et al., 1981; Reeves, 1980; Rossi‐Bernardi et al., 1975; Zwart et al., 1982). These experimental setups while technically precise, were often time‐consuming with a lack of efficacy and with limitations concerning availability and feasibility (reviewed in Zwart et al. [1982]). In the last decade new methods for O_2_ affinity measurements appeared, for example, based on measurements of entire absorbance spectra of samples and controls in a microplate. However, despite providing a high sample throughput, these techniques are not designed to allow recordings of ODCs over the whole range (Nakagawa et al., 2014; Patel et al., 2018).

Currently, the only commercially available instrument for direct ODC determination is the Hemox Analyzer from TCS Scientific Corp. The instrument is constructed as a mono‐cuvette system. Designed as such, one ODC determination (excluding cleaning and other preparative steps) takes about 20–30 min, making it rather impractical for larger amounts of samples or collectives of patients.

Fully automated blood gas analyzer (BGA) instruments are standard for in‐hospital P50 determination. Via single‐point measurement of *P*O_2_ and corresponding *S*O_2_ the P50 is estimated by known algorithms. However, compared to direct measurements (e.g., by the Hemox Analyzer), sensitivity (5%), and specificity (77%) were shown to be low (Huber et al., 2013).

Currently, because of the difficulty and impracticability of measuring the ODC, the determination of P50 values and Hill coefficients plays a secondary role in critical care medicine and hematology. This is regrettable in view of recent discussions (Böning & Schmidt, 2020; Dempsey, 2020; Dominelli et al., 2020) that P50 modulation might well be an underestimated therapeutic target for optimizing O_2_ transport to the tissue, besides modulation of cardiac output and Hb concentration (Lucas et al., 2019; Srinivasan et al., 2017).

We aimed at developing a new method for precise and efficient ODC determination with large series of measurements (*n* = 92) to be accomplished in <30 min. As ODC is supposed to be best measured under pure physiological conditions, the goal was to derive ODCs using whole blood without buffer or other agents.

## 2 MATERIAL AND METHODS

### 2.1 Construction, design, and principle of operation

**FIGURE 1:**
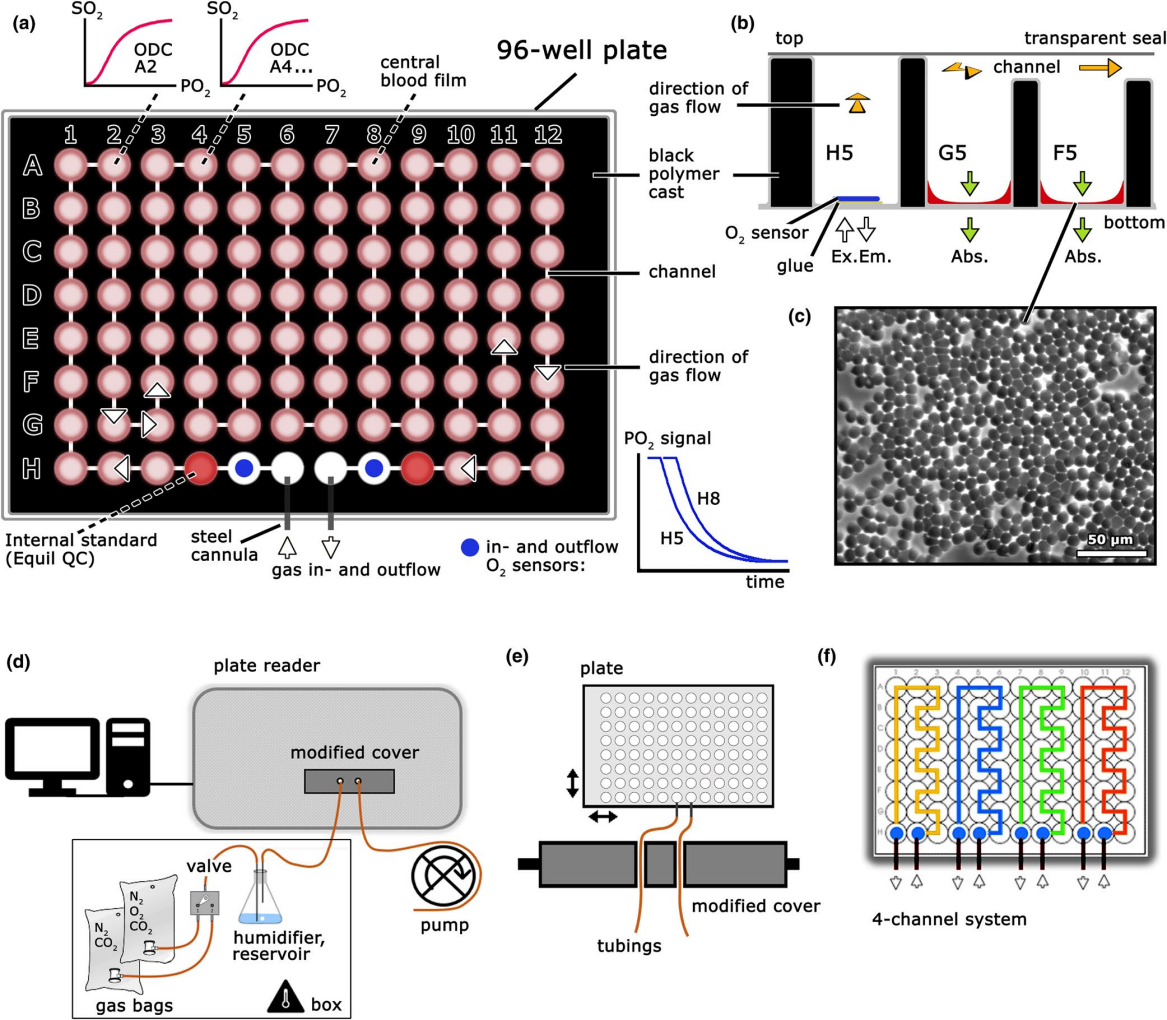
Scheme of the method to determine, in parallel, ODC of small blood samples by a modified 96‐well plate and a microplate reader. (a) ODC plate. All wells are interspaced with a black polymer, and channels milled to form a meandering gas flow system when covered with a transparent seal. Wells are either empty (white) or contain thin films of whole blood (pink), internal standards (red) and two O_2_ sensors (blue) as depicted. Signals of the in‐ and outflow O_2_ sensors (blue line graphs) and two (A2 and A4), out of 92, exemplary ODCs are shown (top). (b) Cross‐section through wells H5‐F5 with details of the polymer cast and the gas channel system, as well as principle of measurements (fluorescence intensity in the bottom reading mode for oxygen partial pressure, *P*O_2_, absorption measurements for oxygen saturation, *S*O_2_). (c) Microscopy of the central blood film showing a 1–2 cell layer of red blood cells over large parts. (d) Entire setup with computer, gas supply, temperature‐controlled humidifying gas reservoir, modified cover, microplate reader (front view), and a peristaltic pump (bottom right). (e) Top‐view of the modified cover allowing a flexible, smooth, and light‐proof connection between the ODC plate and gas supply/outflow. Arrows indicate movements of the plate within the microplate reader during measurements. (f) Depicts an exemplary possibility for modifying the ODC plate with four independent (colors) gas flow systems (= four‐channel ODC plate). ODC, oxygen dissociation curve

A Cell‐Star 96‐well plate (Greiner Bio‐One GmbH) was used. All rear cavities of the plate were cast with a black polymer (colored epoxy resin). After hardening (24 h), the entire top of the plate was milled (1–2 mm in depth, 3 × 3 mm at the channels) to generate a flat, smooth surface with a meandering gas flow channel (Figure 1a,b). Planar O_2_ sensor spots (PreSens Precision Sensing GmbH) were inserted in wells H5 and H8. Wells H3 to H10 along the flow‐through channel were filled with small volumes (15 µl) of whole heparinized blood, forming a stable central and thin (1–2 cell layer, Figure 1c) film of red blood cells (RBC) by applying a particular plating method as described below. Wells H4 and H9 contained the internal standard (Equil QC 463, RNA Medical, see Supplemental Information for its composition). After plating the RBC, the top of the plate was sealed with a transparent, adhesive microplate sealer (Greiner AG), providing unidirectional gas flow along the channels and gas tightness between the wells and the exterior (Figure 1b). Gas in‐ and outflow occurred via steel cannulas fixed in wells H6 and H7. They were connected to a peristaltic pump (outflow) and a gas mixing reservoir (inflow) via small (ID = 1.5 mm) flexible tubes made of gas‐tight Viton (Figure 1d,e). Viton tubes, humidifiers, and the modified cover were placed in a thermostatic box. The *P*O_2_ of the gas mixture at both ports was recorded with the O_2_ sensors, and the gas composition (i.e., *P*O_2_) between all other wells (H4–H9) was calculated by linear interpolation of the shift between both sensor signals (graph in Figure 1a), as described below. *P*O_2_ was determined by fluorescence measurement at the plate bottom and *S*O_2_ by dual wavelength absorbance measurement (see below). By combining the paired *P*O_2_ and absorbance measurements, ODCs for all wells (except H5 and H8 housing the non‐transparent sensor spots) were generated. Examples of ODCs are depicted for wells A2 and A4 in Figure 1a. A typical ODC measurement of the entire plate, comprising up to 90 single ODCs and two ODCs of an internal standard solution, was completed in about 25 min. Moreover, the plate may be modified with multiple, for example, four, independent gas flow channels (= four‐channel ODC plate; Figure 1f). Multiple channel plates allow simultaneous ODC recordings with differing gas constituents (e.g., *P*CO_2_ levels, see below).

By using O_2_ sensors, actual *P*O_2_ is measured, rendering ODC determinations independent of predefined gas mixes or specialized gas mixing devices. Any means of making a gradual change in O_2_ will therefore suffice. In our system, a gradual decline in O_2_ from 20 vol % to 0 vol % (= O_2_ ramp) was started by passing an O_2_‐free gas (pure N_2_ or N_2_ supplemented with CO_2_) through a half‐filled humidifier containing 20% O_2_ (= reservoir, humidifier Figure 1d). This served to supply the air stream with 85.6% relative humidity while generating a presumably exponential fall in O_2_. Calibration of the O_2_ sensors was done with 20 vol % O_2_ (start of the experiment) and 0% O_2_, which was achieved with pure N_2_ bypassing the humidifier at the end of every ODC determination (see below).

### 2.2 O_2_ measurement and calibration

*P*O_2_ determination by the O_2_ sensors in the ODC plate is based on the O_2_‐dependent quench of luminescence caused by a collision of molecular O_2_ and luminescent dye molecules in the excited state (PreSens Precision Sensing GmbH; measuring range is from 0% to 100% O_2_, and precision ±0.01% at 0.21% O_2_, and ±0.1% at 20.9% O_2_). For use in a microplate instrument (Tecan Infinite M200 Pro, Tecan GmbH), we applied the custom fluorescence measuring mode set to 1 flash at 543 nm excitation with an integration time of 60 µs and a 6 µs delay following flash illumination. Emission was recorded at 653 nm. The measured integral of fluorescence lifetime provides a signal that is inversely related to O_2_. Sensor readouts can be converted to O_2_ following a two‐point calibration with an O_2_‐containing and an O_2_‐free gas mixture as follows:

(1) $$O_{2}=\left(\left(I_{0}/I\right)‐1\right)/K$$

where *I*_0_ = fluorescence intensity with zero O_2_ (end of measurement), *I* = fluorescence intensity with O_2_‐containing gas mixture (start of measurement), and *K* = sensor constant (calculated by two‐point calibration for every measurement. *P*O_2_ is known at the start and the end of the measurement, see Equation 2).

Actual *P*O_2_ of moist O_2_‐containing gas mix was calculated as:

(2) $$PO_{2}=\left(P_{\text{atm}}‐\Delta P‐PH_{2}O\right)\times \text{vol}\;\%{\;O}_{2}$$

where ∆*P* is the difference between the current barometric pressure (*P*_atm_) and the pressure in the ODC plate due to suction produced by the peristaltic pump, amounting to ~3 mmHg. Values for *P*_atm_ were obtained from a local meteorological station and vol % O_2_ is known due to the volumetric generation of gas mixtures by gas‐tight syringes. Humidity (*P*H_2_O) was recorded by a data logger (EB‐Logg 80CL‐E, Greisinger GmbH) and sensors placed into tube extensions at the inlet and outlet ports of the ODC plate. Relative humidity values approximate full water vapor saturation (relative humidity = 93.6 ± 1.0%), measured after the outlet port, were slightly higher than those values determined at the inlet port of the ODC plate (85.6 ± 1.6%). This might be caused by water evaporation from the samples. However, evaporative water loss from samples, when calculated or measured directly (see Supplemental Information), amounted to only 0.11 µl per well (or 0.7% of the sample volume).

Sensor readout was allowed to stabilize with the calibration gases for 3 min. These values were taken at the beginning and completion of every ODC determination. Such a routine calibration also served to eliminate potential sensor drifts, which may be caused by slow photodecomposition of the O_2_‐sensitive material (PreSens). Noteworthy, calibration is particularly essential in experiments using various temperatures due to a substantial thermal sensitivity of sensor performance. Temperature in the plate reader is controlled by an inbuilt hot air thermostat providing 37 ± 0.2℃ throughout the measurements.

**FIGURE 2:**
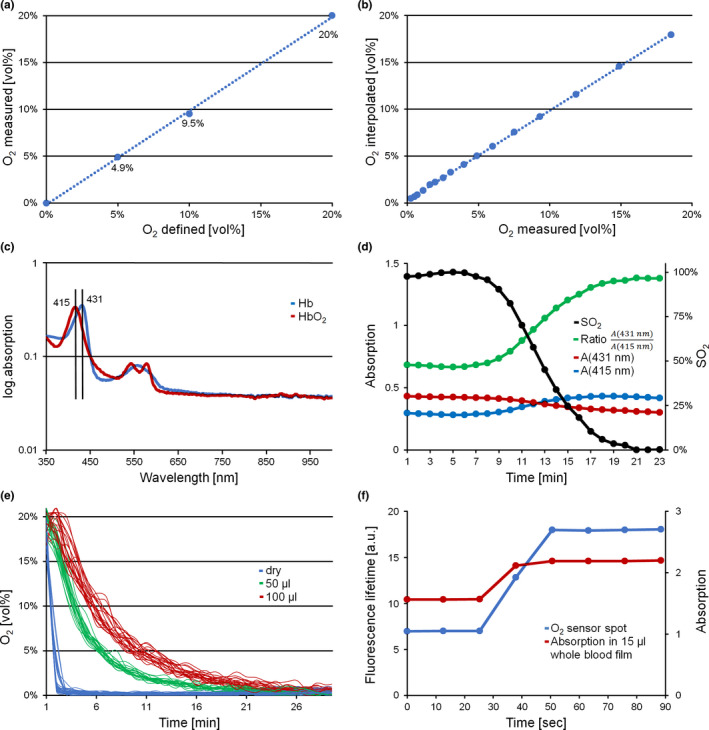
Measurement conditions regarding determination of O_2_ and hemoglobin oxygen saturation with characterization of the respective response times. (a) Measured versus defined O_2_. Numbers in the graph refer to the measured values. (b) Linear interpolation of O_2_ (see Equation 3) as compared to measured O_2,_ shown exemplarily for well G12. (c) Hb absorption spectra as measured in the blood films of the ODC plate. Red line HbO_2_, blue line Hb. Semilogarithmic presentation. (d) Absorption and calculated ratios during an ODC experiment shown exemplarily for well A6. Red (431 nm) and blue (415 nm) lines are raw absorption data, the green line is absorption ratio (431/415) and the black line oxygen saturation, *S*O_2_ (%). (e) O_2_ sensor readout under dry conditions and in various volumes of aqueous solutions (0.9% NaCl), measured in Oxoplate (see Supplemental Information). Gas supply (20% O_2_) was abruptly switched to 100% N_2_. Fast decrease under dry conditions (blue), delayed decrease in sensors submerged in a 50 µl fluid volume (green) and protracted response at 100 µl (red). (f) Response times of the O_2_ sensor spot under dry conditions (blue) and *S*O_2_ of a 15 µl whole blood film (red) at a steep ramp of O_2_ from 20% to 0% (switch to N_2_ at time 30 s). ODC, oxygen dissociation curve

Linearity and accuracy using the two‐point calibration were tested with different O_2_ gas mixtures and by analyzing the two intermediate O_2_ levels (5 vol % and 10 vol %). Deviations between actual and calculated values were low (Figure 2a), thus eliminating the need for non‐linear curve fitting procedures. Accuracy was particularly high at low O_2_ levels (5 vol %), which are close to the expected P50 of a typical ODC (approx. 27 mmHg).

In the single‐ as well as the four‐channel ODC plates we used an O_2_ sensor at the inlet and outlet port of the respective flow‐through channel (Figure 1a,f). This was required due to the temporal offset between both sensor signals when using a gas ramp of slowly decreasing *P*O_2_ over a time period of ~20 min. By assuming undisturbed gas flow along the channels, the actual gas composition between all other wells can be approximated by linear interpolation, as exemplified by Equation (3) for an arbitrary intermediary well (e.g., E7, which is at position 49 in the gas flow system):

(3) $$PO_{2}=PO_{2\text{start}}+\left(49/93\right)\times \left(PO_{2\text{end}}‐PO_{2\text{start}}\right)$$

Accuracy of this approximation was tested using three additional sensor spots inserted at different distances along the flow‐through channel while running an O_2_ ramp. Interpolated versus measured values for one sensor spot are shown in Figure 2b. The mean Δ*P*O_2_ between the two sensors is 8.1 ± 2.6 mmHg (*n* = 20) at minute 15, at which the data points are close to 50% *S*O_2_. The difference is due to the fact that sensor 1, at the inlet port, is receiving a reduced O_2_ before sensor 2 at the outlet port.

### 2.3 SO_2_

Absorbance spectra of oxygenated (HbO_2_) and deoxygenated hemoglobin (Hb) are shown in Figure 2c. For the *S*O_2_ calculations of samples and the internal standard we used the ratio of background corrected absorption at the Soret bands as indicated (431/415 nm) (Reeves, 1980). Typical changes in these values during a deoxygenation protocol are exemplarily shown in Figure 2d. Ratiometric dual wavelength measurements were used to compensate for RBC film volume changes in the optical path, caused by pipetting artifacts or changes in film thickness during the measurements (Figure 2d).

### 2.4 Combined *P*O_2_ and *S*O_2_ measurements

Near equilibrium conditions regarding measurements of *P*O_2_ and *S*O_2_ are essential in our approach. Response time of the O_2_ sensors under dry conditions is in the range of seconds (<6 s, according to manufacturer technical data) and mainly determined by gas diffusion through the sensor material (PreSens). Similarly, the rate‐limiting step in O_2_ binding to Hb is molecular diffusion of O_2_ through the liquid phase. This may be of particular importance when measurements are performed under non stirred conditions, as in our case. As shown in Figure 2e, the increase in fluid volumes per well increased response times dramatically. We thus aimed to create a blood film with as small a volume as possible: 15 µl of whole blood per well proved to be optimal. Volumes exceeding 30 µl produced absorbance values out of instrument range (>4), and values below 10 µl led to breaks in the blood film, probably due to strong meniscus forces (data not shown). Thus, we compared reaction times of the sensor spots and *S*O_2_ following a single steep change in *P*O_2_ (Figure 2f). As demonstrated, the time lag of both signals was similar and in the range of 20 s (due to the volume in tubes from valve to oxygen sensor and blood film). This is very short compared to the duration of an O_2_ ramp, lasting about 20 min from full Hb oxygenation to deoxygenation. We thus assumed that our measurements obey quasi‐static equilibrium conditions, that is, that *P*O_2_, measured in the air‐space, actually reflects *P*O_2_ inside the erythrocytes (as discussed by Reeves [1980]).

### 2.5 Curve fittings and parameter calculations

**FIGURE 3:**
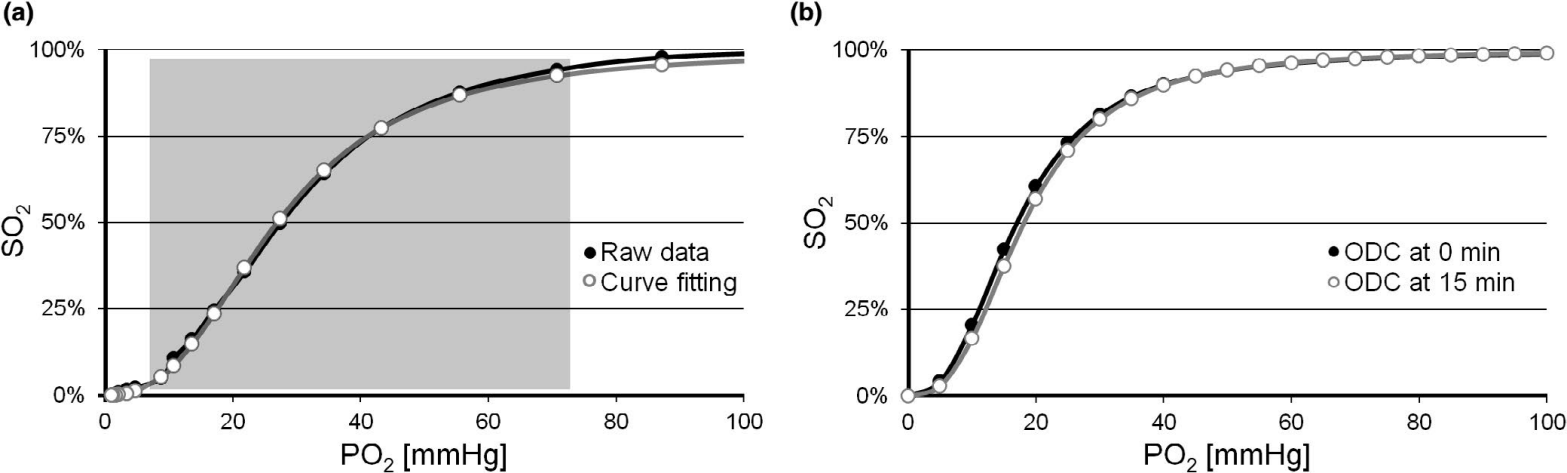
Raw data of ODC determination, regression analysis and shift of the ODC by pH change during ODC recording. (a) Raw data (black) and the ODC after curve fitting (white) by nonlinear regression analysis using the Hill function and least square method. The continuous Hill function was fitted using the data points between 5% and 95% *S*O_2_, as indicated by the shading. (b) Right‐shift in ODC (white) within 15 min compared to ODC at time zero (black), resulting in a calculated ΔpH of −0.05. ODC, oxygen dissociation curve

An exemplary ODC measurement (raw data) and curve fitting is shown in Figure 3a. Raw data points were fit with a modified version of the Hill function (equation 0 in Goutelle et al. [2008]) using the least‐square method (Microsoft Excel), where C = P50, *α* = the Hill coefficient and dyshemoglobins the fraction of non‐functional Hb, for example, methemoglobin (MetHb) and carboxyhemoglobin (COHb), according to Equation (4). The fraction of dyshemoglobins was obtained by BGA and considered for the measurements in the Application Section only, subtraction of the fraction of dyshemoglobins is optional. Note that, besides the provided ones, any proper equation or procedure for curve fitting may be used.

(4) $$SO_{2}=\left(\left(100\%‐\text{Dyshemoglobins}\right)\times PO_{2}^{\alpha }\right)/\left(C^{\alpha }+PO_{2}^{\alpha }\right).$$

### 2.6 Blood collection and storage conditions

Venous blood from one donor (S.W.) was withdrawn by venipuncture with 5.5 ml lithium‐heparin vacutainers and stored on ice (= time zero). Effects of storage were evaluated via repeated measurements of aliquots in a BGA (ABL 800 flex, Radiometer Medical Aps). For 8 h plasma pH, MetHb and COHb did not change, whereas potassium levels and lactate increased, glucose and the anion gap decreased (see Supplemental Information). Despite these moderate changes, blood samples were usually measured promptly after collection (1–4 h). Hemolyzed blood was prepared with two freeze‐thaw cycles and centrifugation.

### 2.7 pH measurements

To estimate the pH drift in the blood film during an ODC recording two different and independent approaches were used (*S*O_2_ determination at fixed *P*O_2_ and BCECF fluorescence microscopy). Due to the high complexity of the experiments and the impossibility of an implementation into the ODC recording protocol, these pH measurements were only performed once.

*S*O_2_ determination at fixed *P*O_2_: pH changes were measured indirectly using O_2_/N_2_/CO_2_ mixtures that correspond to the beforehand measured P50 (at constant *P*O_2_) of the same samples. Any change in the measured *S*O_2_ should therefore correspond to a shift in pH. In a regular ODC recording the region of interest for ODC curve fitting and P50 calculation is about 15 min after begin of the measurement. Figure 3b demonstrates the resulting right‐shifted ODC after 15 min that amounts to a calculated pH drift of ∆pH = −0.059 (plasma Bohr coefficient of −0.48 was used for calculation, for details see Supplemental Information).

BCECF fluorescence microscopy: In the blood film plated into the wells of the ODC plate and exposed to a stream of humidified gas mix at 37℃, 20 vol % O_2_ and 5.6 vol % CO_2_, a plasma pH drift of ∆pH = −0.05 was recorded within 15 min (Figure S2). These measurements were performed using the fluorescent pH indicator BCECF, free acid (Molecular Probes Inc.), diluted 1/5 in whole blood, and a fluorescence microscope (see Supplemental Information).

Both approaches, *S*O_2_ determination at fixed *P*O_2_ and BCECF microscopy, revealed a pH shift of <0.06, which is to be expected when using whole blood without supplementation of artificial buffers. However, whole blood may be investigated with any appropriate buffer in the ODC plate, if necessary. However, dilution with buffer solution should not exceed a ratio of 1:10 as absorbance values for the blood film become critically low (data not shown). In a physiological or clinical investigation, it is recommended to perform BGA of aliquots of the same blood samples prior to the ODC measurements.

### 2.8 Plating of RBC and film stability

We performed a series of trials to find appropriate measuring conditions, mostly concerning improvements in blood volume to surface area relationship, and to guarantee high reproducibility of the blood films. The following proved to be optimal: 15 µl of whole blood was pipetted into the corners of the wells. As this volume is not sufficient to be spread in a continuous layer, a piston (stainless steel rod) in the dimension of the wells (clearance 0.2 mm) and flat bottom was used to distribute the blood droplet over the entire well bottom. The steel rods were applied manually at controlled low force (0.32 N/well), without rotation, once (= a single brief contact) and just in order to distribute the sample volume. Once spread, the formed blood film had a central thickness of 1–2 RBC and was stable for at least the duration of one ODC measurement, provided humidified air was supplied throughout. This was confirmed by microscopic analysis, also demonstrating occasional Rouleaux formation (Figure 1c and Figure S3).

### 2.9 Gas mixtures and quality control

Pure gases (O_2_, CO_2_, N_2_; all from Linde GmbH) were mixed volumetrically using gas‐tight syringes. The defined gas mixtures were stored in gas‐tight bags (Restek Corp.). The accuracy of the prepared gases was confirmed using a conventional gas measuring module (Dräger Vamos, Version 03.07, Dräger GmbH). The actual gas ramp (20 to 0 vol % O_2_) used to measure ODC was generated by slow displacement of a defined volume (120 ml) of 20 vol % O_2_ and 5.6 vol % CO_2_ in the humidifying reservoir by continuous flushing with N_2_ and 5.6 vol % CO_2_ (see above and Figure 1d) over a time period of 20 min.

### 2.10 Statistics and data representation

Student's *t*‐test (two‐tailed) was used to compare means. Microsoft Excel was used for data collection, calculation and analysis. *p* < 0.05 was considered statistically significant. Results are expressed as arithmetic mean ± standard deviation (SD). One experiment is defined as one measurement of an entire ODC plate.

### 2.11 Intra‐assay variability

**FIGURE 4:**
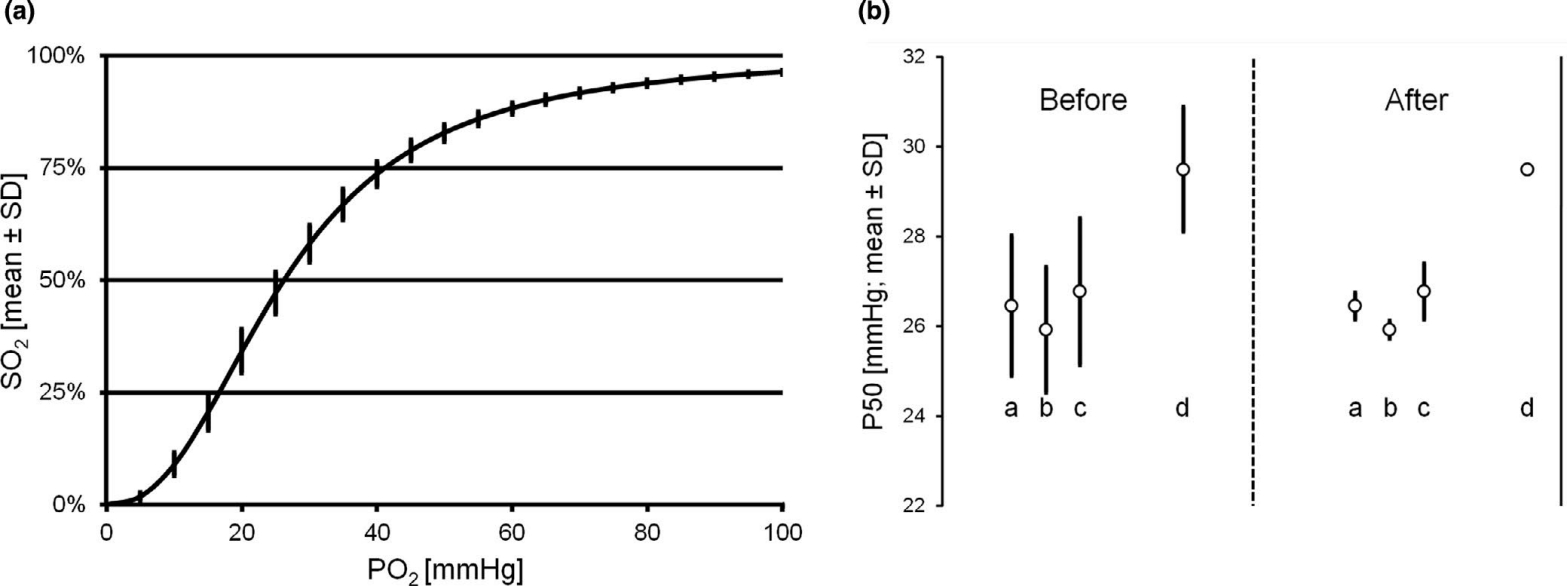
Analysis of intra‐ and inter‐assay variability of the ODC method. (a) ODC intra‐assay variability (mean ± SD). Averaged ODC of a whole plate (*n* = 92). (b) ODC inter‐assay variability of three experiments with whole blood samples from three different persons (a, b, and c) and the internal standard solution (d) before and after correction with the internal standard solution. ODC, oxygen dissociation curve

Within one experiment (whole blood from one donor, S.W.; *n* = 92 wells) mean P50 was 26.5 ± 1.61 mmHg and coefficient of variation ± 0.06. SD was highest in the steep part of the curve, levelling off in the flat, asymptotic ranges (Figure 4a). Overall mean SD (comprising all data points in Figure 4a) was ±0.015 mmHg. A heat map of P50 distribution within one ODC plate is shown in Figure S5. Considering intra‐assay variability, an averaging of individual measurements (wells), either in a structured or random manner, is advisable. For example, the intra‐assay variability (SD) further decreased to ±0.86 when averaging three, or to ±0.53 when averaging the results of six adjacent wells.

### 2.12 Inter‐assay variability

One operator performed three consecutive ODC measurements using blood samples from three colleagues. Per experiment and person and by averaging three wells (see above) we obtained 24 ODC results, 72 in total. Interassay variability was 0.52 (mean of inter‐assay SD in three persons) before and 0.27 after correction with the internal standard solution (Figure 4b). Since several factors can cause variance in consecutive ODC measurements (Lucas et al., 2019), correction of inter‐assay variability with an internal standard improves both reliability of data and robustness of method.

### 2.13 Comparison with Hemox Analyzer

The Hemox Analyzer can be considered a standard instrument for ODC measurements and P50 calculations (Patel et al., 2018). A comparison of our results with those achieved with this instrument was performed using Equil QC 463, level 2, a highly standardized sample with a P50 that is expected to be stable. Unpublished reference data using Equil QC 463 in a Hemox Analyzer were kindly provided by Dr. Jason Acker, University of Alberta, Canada (Acker et al., 2014). Following the manufacturer's instructions and Acker's protocol, a 100‐fold dilution of Equil QC 463 in a solution of Hemox buffer, albumin and anti‐foaming agent (TCS Scientific Corp.) would have been necessary. However, as our method works with thin films (1–2 cell layers), such a strong dilution is not applicable due to low absorption signals close to zero. We used a 10‐fold dilution instead, but strictly followed the protocol in all other aspects.

In three runs, 216 ODC of diluted Equil QC 463 were recorded and corrected with our internal standard solution, as described above. Defining one result as the mean of three independent wells in one plate, 72 P50 values were calculated and showed a mean of 25.5 ± 1.48 mmHg. This result is slightly lower than that provided by J. Acker, namely a mean of 26.4 ± 1.33 mmHg (*n* = 118; *p* < 0.001).

This deviation might be explained by the properties of the buffer systems used (Hemox buffer, albumin, and antifoaming agent). In addition, our experiments revealed a concentration‐dependent shift of the P50 towards higher levels at low Equil QC 463 concentrations in Hemox buffer. To explain this shift, samples of different Equil QC 463 concentrations were analyzed with a high‐end BGA. The pH was found to be lower (7.276 at 100× dilution and 7.294 at 10× dilution; *n* = 4, *p* < 0.01) and Cl^−^ to be higher (135 mmol/l at 100× dilution and 131.25 mmol/l at 10× dilution; *n* = 4, *p* < 0.01) at higher dilutions. In summary, a higher pH, a lower Cl^−^‐ concentration, and different concentrations of albumin and antifoaming agents in our system may account for the slight difference in P50.

## 3 APPLICATION

### 3.1 Hemolyzed blood

**FIGURE 5:**
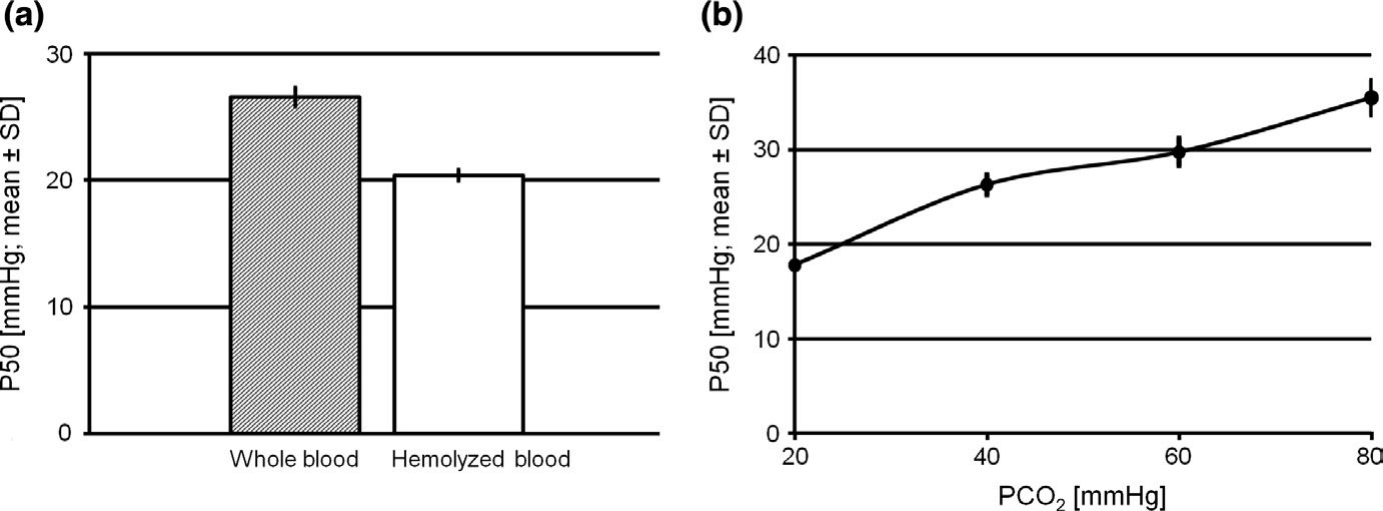
Examples of application of the ODC method. (a) P50 (mean ± SD) of whole (shaded bar) versus hemolyzed blood (white bar). (b) CO_2_ Bohr effect. P50 (mean ± SD) of whole blood samples from three different persons exposed to four different levels of CO_2_ at a time using the 4‐channel plate. ODC, oxygen dissociation curve

P50 of untreated, venous whole blood (S.W.), and P50 of the same sample hemolyzed in two freeze–thaw cycles are compared in Figure 5a. The respective values were lower after hemolysis (20.4 ± 0.46 mmHg vs. 26.6 ± 0.76 mmHg; *p* < 0.001), while Hill coefficients remained constant (2.67 ± 0.07 vs. 2.65 ± 0.10; *p* = 0.66). Hemolysis by freeze–thaw cycling and centrifugation exposes Hb to mixed intra‐ and extracellular conditions. The decrease in P50 might be explained by partial liberation of phosphate groups, particularly 2,3‐BPG, from the Hb binding sites together with dissipation of the cellular pH gradient, both causing an increase in O_2_ affinity and a left‐shift in the ODC (Imai, 1982). Thus, it is feasible to use hemolyzed blood samples for ODC analysis, as long as the factors leading to the lower P50 values in unphysiological conditions are considered.

### 3.2 CO_2_ Bohr effect

We used the four‐channel ODC plate (Figure 1f) to study the effect of different *P*CO_2_ levels in unbuffered samples (CO_2_ Bohr effect). Venous whole blood samples from three authors and the internal standard solution were equally distributed on the plate and exposed to four different gas mixes (*P*CO_2_ = 20, 40, 60, and 80 mmHg) simultaneously. The gases were supplied in separate gas collection bags and humidifying reservoirs, whereas the outflow tubes were connected in parallel to the same peristaltic pump. The CO_2_ Bohr effect is shown in Figure 5b. The non‐logarithmic plot demonstrates a significant non‐linearity with a pronounced effect, particularly in the simulated hypocapnic situation (*P*CO_2_ = 20 mmHg).

## 4 DISCUSSION

### 4.1 Methodological approach

We present a novel high‐throughput method for ODC determination utilizing a conventional fluorescence microplate reader.

Data show that *P*O_2_ measurements can be realized with adequate accuracy and precision using commercial O_2_ sensors inserted into a 96‐well plate and with fluorescence lifetime read out on a conventional microplate reader. This simplification of the sensor readout allows O_2_ sensors to be combined with other additional measurements such as absorption. Combining *P*O_2_ measurement in the air space with dual wavelength absorption measurements in samples of whole blood, plated as thin films in adjacent wells, *P*O_2_ and *S*O_2_ can be measured side by side because both parameters have almost identical response times. By continuously decreasing O_2_ content over time, measurements executed every minute give the record of ODC. It is sufficient to use two O_2_ sensor spots with linear interpolation of *P*O_2_ over all wells along the meandering gas‐tight gas flow system for measuring up to 92 individual ODCs simultaneously. Depending on the target, either to increase the number of samples per plate or to improve the precision of results, the average can be taken from three or any number of single wells/measurements. Precision can be further improved and inter‐assay variability corrected by using appropriate controls or internal standards with defined O_2_ affinities, for example, Equil QC 463, in the same experiment. Blood samples can be measured without artificial buffers to mimic physiological conditions as accurately as possible, for example to analyze the combined CO_2_ Bohr effect as shown above. However, at 37℃ a pH drift due to RBC metabolism needs to be considered (see Supplemental Information). Blood samples can also be measured in any buffered system or even in hemolyzed conditions.

Techniques for ODC determination (Duvelleroy et al., 1970; Nelson et al., 1981; Reeves, 1980), as mentioned in the introduction, either rely on stirring buffered and highly diluted blood samples or use other sophisticated approaches that have notable precision but a general lack of sample throughput. With the presented method we attempt to combine both advantages in order to provide a high sample throughput that is not at the expense of precision.

Our method, in contrast to, for example, the Hemox Analyzer, allows parallel ODC determination of a large number of samples per experiment using a standard laboratory instrument. Microplate readers are widely used multi‐purpose instruments that are available in most laboratories. After making some smaller modifications in these instruments, in particular in the gas in‐ and outlets, this method can be easily implemented in nearly any existing system. This could increase the ability to determine ODCs in labs worldwide and even make it a routine procedure. Recently published methods with high‐throughput design are semi‐quantitative and do not record the slope of the curves (Nakagawa et al., 2014; Patel et al., 2018). ODCs that are recorded by several data points over the whole range of the curve, however, would actually be needed to reveal high O_2_‐affinity hemoglobinopathies with impaired cooperativity (Mangin, 2017) or any structural or functional pathology in Hb in general that is not easily detected with an algorithmic one‐point estimation of P50.

Finally, the simultaneous measurements of different gas mixtures give an unprecedented advantage over existing methods using mono‐cuvette systems since frequent confounders (e.g., barometric pressure changes, temperature fluctuations, and most prominently different sample storage times) can be excluded. To our knowledge, no commercially available method is presently able to measure ODC in different gas compositions in one experiment.

### 4.2 Relevance

The poor availability of methods for P50 determination and particularly methods for determination of the ODC in order to detect variations in the slope of O_2_ binding, limits diagnostic use (Mangin, 2017). In hematology, for example, P50 can be used as a predictive parameter of Hb functionality before complex molecular biological testing is attempted in patients with erythrocytosis and suspected hemoglobinopathy (Mangin, 2017). In the diagnostic evaluation of polycythemia, ODC determination is a key step (Bento et al., 2014; Mangin, 2017; Orvain et al., 2017; Shepherd et al., 2019) and part of recent guidelines (McMullin et al., 2005, 2007). A survey conducted at French and Belgian hematology centers investigated the use of phenotypic determination of high O_2_‐affinity Hb as a pre‐test. Due to the limited availability of the Hemox Analyzer instrument, this approach is used at only half of the laboratories (Orvain et al., 2017). Monitoring P50 and determination of ODC could also be useful to optimize treatment and support in intensive care patients and in other clinical settings (Morgan, 1999; Srinivasan et al., 2017). For example, low 2,3‐BPG levels were shown to be a main parameter affecting ODC in critically ill patients, and more routine determination of P50 would be required to allow P50 modifications in such cases (Morgan, 1999). In patients with sickle cell disease, Hb in the relaxed state (oxygenated) seems to be less vulnerable to sickling and therefore left‐shifting the ODC is discussed as a therapeutic target (Nakagawa et al., 2014).

ODC should be determined from fresh blood, which means these measurements should be available at the same centers where the blood is drawn. This is not the case at present in a great majority of hospitals or clinical centers that instead rely on P50 estimations by BGA. For diagnostic use and monitoring, a method with high throughput and low price is advantageous, and comparison of several blood samples with a standard Hb solution is beneficial for precise and reliable detection of P50 alterations. By integrating the proposed method into clinical diagnostic or therapeutic monitoring procedures, information about O_2_ affinity and thus O_2_ transport can be gathered in a reasonable timeframe. For example, it would be possible to measure ODC and P50 of up to 30 patients within 25 min with minimal effort since experiment handling is simple and analysis runs semi‐automatically. This would be helpful for clinical trials since the method permits various samples to be determined simultaneously, which optimizes study protocols and improves data comparability.

Besides the clinical use, a robust, affordable method with high throughput would be beneficial for many different applications in hematology, pharmacology, physiology, or zoology.

### 4.3 Limitations

Absolute values of pH cannot be measured in such thin films of blood as used in our experiments. We therefore recommend simultaneous BGA or pH measurements of aliquots (see chapter “Section 2.7”). Corrections with respect to standard pH (7.40) can then be made using the Bohr coefficient, considering its *S*O_2_ dependency (Böning et al., 2014).

*P*O_2_ is measured by dry O_2_ sensors and not directly in the samples. However, in chapter “Section 2.4” this issue is comprehensively discussed and a possible delay of O_2_ change in the blood films should be negligible.

Standard deviation (intra‐ and inter‐assay variability) in P50 and *P*O_2_ measurements also has to be considered. We therefore strongly recommend averaging of individual measurements (wells), the use of an internal standard solution (e.g., Equil QC 463) and/or appropriate controls in separate wells.

Measurements of *P*O_2_ and *S*O_2_ are not continuous. Nonetheless, the number of single data points (~10) is sufficient to describe the sigmoidal shape of the ODC.

Humidity of the gases should be as high as possible to minimize the rate of evaporation from samples.

A direct comparison of our method with other techniques (see chapter “Section 2.13”) is limited, due to the inherent differences in experimental design.

## DISCLOSURE

The authors declare no conflicts of interests. Patent protection for the ODC plate, to be shared by the Medical University of Innsbruck and Eurac Research as applicants, and with S.W. and T.H. as the inventors, is pending (European Patent Office, EP20204184.4).

## AUTHOR CONTRIBUTIONS

Conceptualization, S.W. and T.H.; methodology, S.W., N.M. and T.H.; validation, S.W., M.S., H.B., G.S., H.G., N.M., T.H.; formal analysis, S.W. and T.H.; writing—original draft preparation, S.W. and T.H.; writing—review and editing, S.W., M.S., H.B., G.S., H.G., N.M., T.H.; visualization, S.W. and T.H. All authors have read and agreed to the published version of the manuscript.

## Supporting information



Data S1, Figs S1–S5
