# Supplementary material for: High‐throughput determination of oxygen dissociation curves in a microplate reader—A novel, quantitative approach
Source: Physiol Rep. 2021 Aug 24;9(16):e14995. doi: 10.14814/phy2.14995 (PMC8383715; doi:10.14814/phy2.14995)
Supplement: Supplementary file 1 — Data S1, Figs S1–S5 [file PHY2-9-e14995-s001.docx]

**Supplemental Information**

**Settings for the microplate reader**

The temperature control system in the Tecan microplate reader (Tecan Infinite M200 Pro) was used for thermal stabilization of the entire instrument (37 °C or other requested levels). The kinetic cycle function was set to one measurement of the ODC plate every minute, starting with fluorescence and immediately followed by absorption. Fluorescence lifetime was measured in the bottom reading mode with λ = 543 nm and λ = 653 nm for emission. Lag time was set at 6 µs and integration time at 60 µs. Absorption was recorded for wavelengths 415 nm and 431 nm.

**O_2_ diffusion in liquid measurement using Oxoplate (PreSens)**

Oxoplate is a 96-well microplate whose wells are impregnated with O_2_-sensing material. To determine the time of diffusion in different volumes of sodium chloride, we modified such an Oxoplate as explained in the Methods section to create an ODC Oxoplate with a gas flow system.

**Storage of blood samples on ice**

Fresh blood must be stored on ice until use. Nevertheless, some changes in blood parameters may occur (are inevitable) over time and need to be considered. After blood withdrawal (S.W.), a baseline aliquot, an aliquot after four hours and a further one after eight hours of storage were analyzed and compared (Fig. S1A) using a high-end BGA instrument. Whereas pH remained nearly constant with regard to PCO_2_, particularly if PCO_2_ was adjusted by gas composition in the ODC experiment, a decrease in glucose and an increase in potassium level are some metabolic changes noticed in the period observed. The difference in strong ions (sodium minus chloride) is shown. Major changes in these parameters occurred between hours 4 and 8. Fig. S1B illustrates changes in dyshemoglobins. MetHb indicates oxidation of the iron atom from ferrous (2^+^) to ferric (3^+^) form and thus the impossibility to transport oxygen. COHb indicates carboxyhemoglobin (CO bound to Hb) and impairment of oxygen transport.


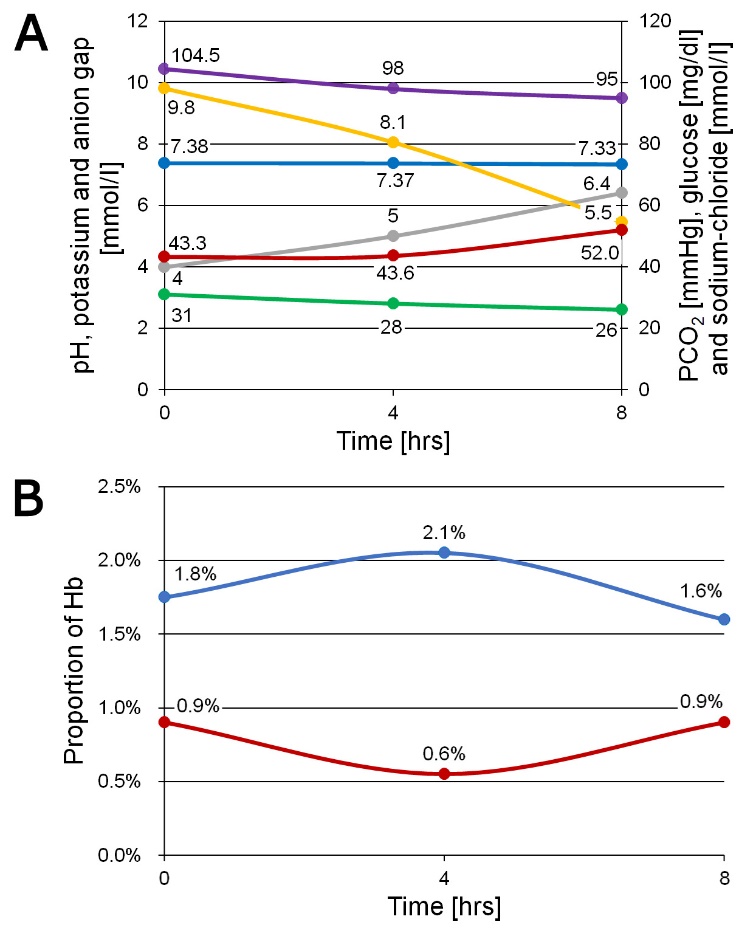


***Fig. S1:*** *Change in blood metabolic parameters (A) and dyshemoglobins (B) during storage on ice (0 °C). Sodium-chloride is the difference of the two ion concentrations.* ***A:*** *Blue line indicates pH, grey line potassium and yellow line anion gap (all left axis); Red line indicates PCO_2_, purple line glucose und green line the difference between sodium and chloride (all right axis).* ***B:*** *COHb (blue line) and MetHb (red line).*

**pH changes during an ODC experiment in unbuffered film of red blood cells**

**
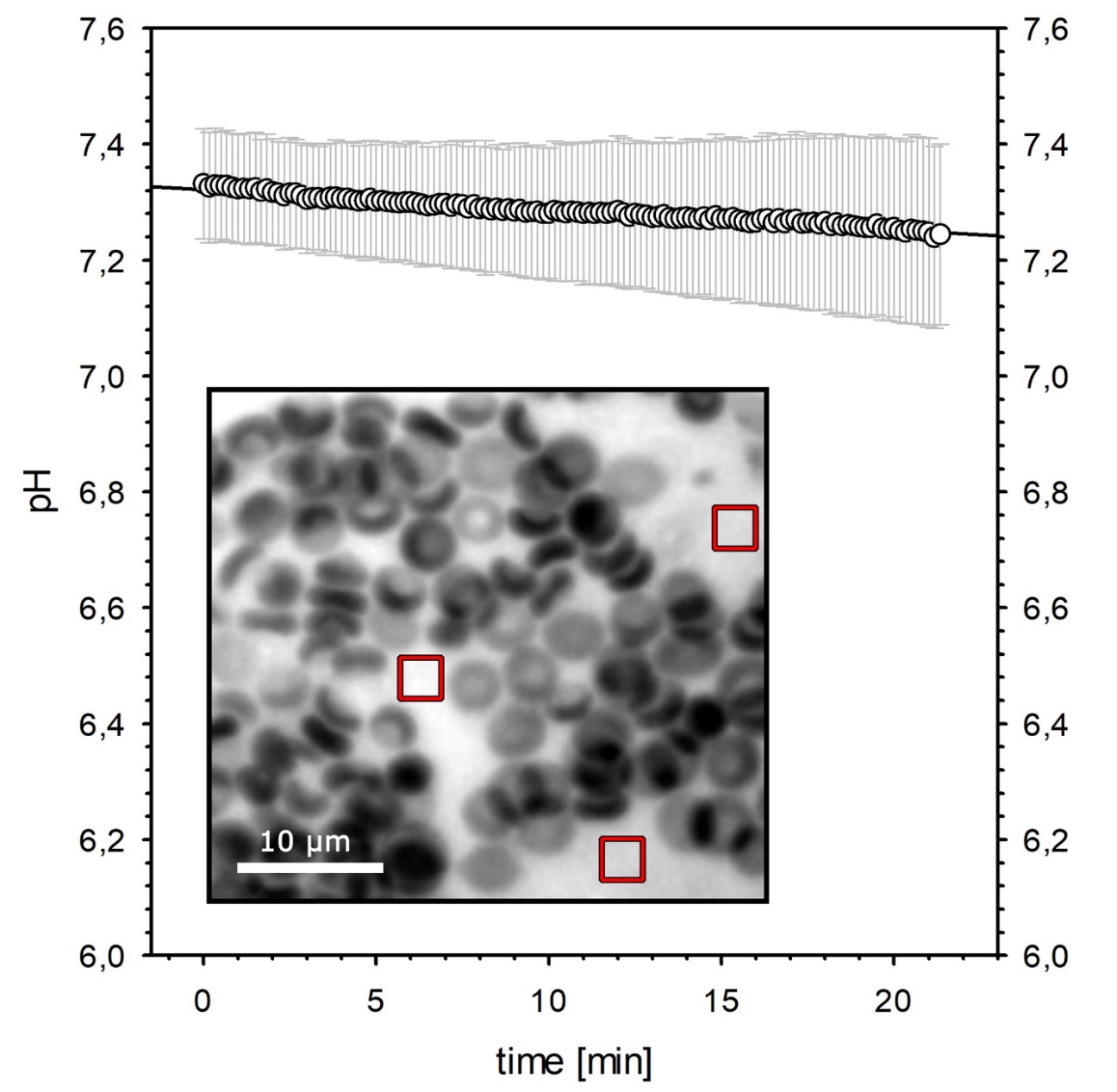
**

***Fig. S2:*** *pH measurements in thin films of whole blood. Thin films of RBC from heparinized samples were plated in 96-wells as described (Material and Methods). In its central part, the film had a thickness of one to three cell layers. During the duration of a typical ODC measurement (20 min), pH decreased linearly (r^2^ = 0.97) by 0.069 units or by 0.052 units in 15 min, the critical time sensitive for P50 determination. Experiments were performed as follows: to whole blood we added 20 % (v/v) pH-sensitive BCECF (free acid; Molecular Probes Inc., USA). High dye concentration was required to obtain reasonable fluorescence intensities in the regions (rectangles) devoid of erythrocytes, several nl by volume. The mixture (15 µl) was plated into the wells and superfused with 5 % PCO_2_ in humidified, ambient air at 37 °C. Fluorescence measurements were performed at 500 nm and 440 nm excitation using an Axiovert S100 TV microscope (Zeiss) equipped with a Polychrome V monochromator (TILL Photonics, Germany), a DAPI/FITC/Texas Red Tripleband dichroic filter set (Chroma, USA), a cooled 12-bit CCD camera (PCO-Sensicam, Germany) and a* *LD Achroplan 40x NA 0,6 long-distance objective (Zeiss). Analysis was made in regions of interest (rectangles) to exclude spectral overlap with Hb. Calibration of BCECF ratio calculations (background corrected intensities at 500/440 nm) relative to pH was made separately in buffered solutions (HEPES) deposited as thin layers onto glass coverslips. The shown micrograph was acquired with a Plan-Apochromat 100× NA 1.4 oil objective (Zeiss) at 500 nm excitation and demonstrates BCECF fluorescence (bright) and dye exclusion from RBC. Number of experiments = 9, arithmetic means ± SD are shown.*

SO_2_ determination at fixed PO_2_: A sample of unbuffered whole blood (S.W.) was exposed to a continuous gas flow with a constant gas composition (O_2_ = 25 mmHg, CO_2_ = 40 mmHg) for 25 min. During a customary ODC experiment P50 is recorded at time point ~ 15 min. Using a single-point curve fitting, P50 was estimated at minute 1 and minute 15. The ODC right-shift was ∆P50 = +1.1 mmHg. Using the plasma Bohr coefficient of -0.48, a corresponding pH drift of -0.059 was calculated (Mairbäurl H, and Weber RE. Oxygen transport by hemoglobin. Compr Physiol 2: 1463-1489, 2012.), corresponding to the pH measurements shown above.

**RBC film stability**

Blood (15 µl) was squeezed over the well bottom using flat-bottomed rods made of polished stainless steel (clearance to well border 0.2 mm). This was the prerequisite for creating a preferably thin but still adherent layer of RBC in the center of the wells. Microscopy confirmed a film thickness of one to two cell layers (Fig. S2). Films were stable for at least the duration of an ODC measurement, with little or no movement of the suspended RBC. Occasionally, and particularly at the very center of the wells, RBC show their characteristic behavior of forming rolls of coins (Rouleaux formation, Fig. S3). Factors leading to Rouleaux formation were not investigated. Degraded RBC like echinocytes were seen to the same low extent as in freshly drawn blood. No changes in RBC morphology were detected before or after ODC determination. No influence on ODC determination was observed.

**
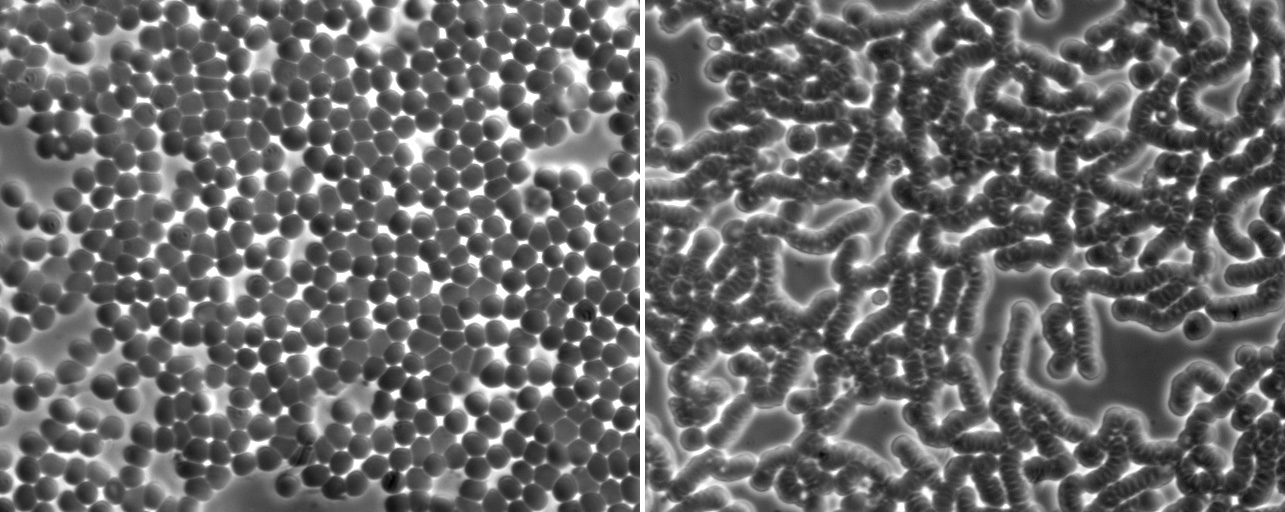
**

***Fig. S3:*** *RBC films without and with Rouleaux formation. Images acquired with an inverted microscope (Axiovert, Zeiss), LD Achroplan 40x objective (Zeiss) and Imago 12-bit CCD camera (PCO-Sensicam, Germany) using phase contrast.*

**Pre-test equilibration phase for the experimental setup**

To guarantee stable conditions during an ODC measurement, the environmentally controlled box (EC-Box) was established. This EC-Box is temperature-controlled and contains the above-described experimental setup (e.g., humidifier, tubes and valves). Using dry gas mixes necessitates an extensive pre-test equilibration phase to ensure stable humidity conditions in tubes and other parts of the experimental setup. While a stable temperature level is reached and maintained after only 30 minutes, asymptotic approximation to a high level of humidity requires up to 2 hours (Fig. S4). Once the moisture content in the tubes has reached this high amount of humidity, temporary disconnection of the humidifier first leads to rapid dehumidification, then rapid re-humidification, reaching this high level of humidity again within few minutes. This indicates that the experimental setup, after the pre-test equilibration phase, is a quick-reacting system, able to ensure certain environmental conditions.

**
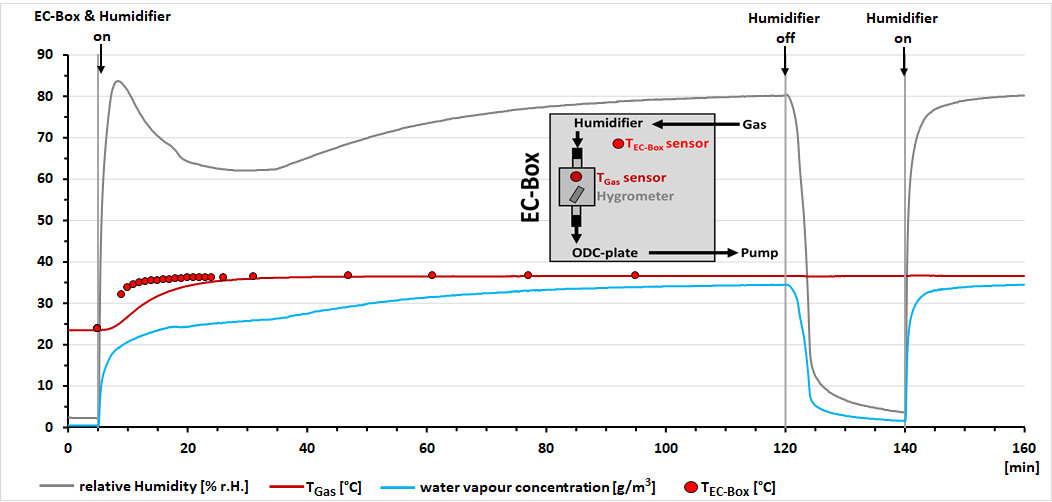
**

***Fig. S4:*** *Pre-test equilibration phase for the experimental setup, e.g., the environmentally controlled box (EC-Box), gas humidity and gas temperature. X-axis displays time in minutes.*

**Estimation of evaporation within the ODC plate**

Relative humidity being lower at the inlet port (85.6 %) compared to the outlet port (93.6 %) raises concerns about evaporation within the ODC plate during an ODC experiment. Using these values, the maximum water vapor content of air at 37 °C (43.9 g/m^3^), and the gas perfusion velocity, the resulting evaporation and thus fluid loss in the ODC plate was calculated and amounted to 3.9 mg. In a second approach evaporation was measured gravimetrically by weighing the ODC plate before and after an ODC experiment. Weight loss of a plate with 84 blood samples varied between 3 and 21 mg, with a mean (n = 4) of 9 mg, denoting a mean loss of 0.11 µl per well (sample), or a sample volume reduction by 0.7 % (from 15 to 14.89 µl/well) during an experiment. Both approaches show minor effects of evaporation on the blood films, in particular when considering that P50 is recorded at half time of an ODC experiment and thus evaporation would amount only half of the here reported values either.

**Internal hemoglobin standard solution**

Equil QC 463 (RNA Medical, USA) is a standard bovine hemoglobin solution containing electrolytes, glucose and lactate: pH = 7.40; Na^+^ = 136 mmol/l; K^+^ = 5.0 mmol/l; Cl^-^ = 98 mmol/l; Ca^2+^ = 1.10 mmol/l; Mg^2+^ = 0.60 mmol/l; Glucose: 100 mg/dl; Lactate = 1.3 mmol/l; COHb = 1.5 %; MetHb = 1.5 %.

**Mapping of P50 within one ODC plate**

Mapping of P50 within the plate is shown in the Figure below, additional heat mapping should help to identify possible patterns. Mean and SD in this exemplary experiment was 25.2 ± 1.2 mmHg. Note that row G is excluded because it contained the internal standard (Equil QC 463). Blood samples from 1 donor were used.

|  | **1** | **2** | **3** | **4** | **5** | **6** | **7** | **8** | **9** | **10** | **11** | **12** |
| --- | --- | --- | --- | --- | --- | --- | --- | --- | --- | --- | --- | --- |
| **A** | 26.3 | 25.2 | 25.3 | 27.2 | 23.4 | 23.2 | 23.9 | 22.9 | 25.6 | 25.7 | 22.9 | 24.3 |
| **B** | 25.5 | 25.0 | 26.0 | 25.2 | 22.6 | 24.6 | 23.6 | 22.2 | 24.9 | 24.2 | 25.7 | 24.5 |
| **C** | 24.9 | 25.9 | 25.4 | 25.8 | 24.3 | 24.5 | 25.1 | 24.2 | 25.0 | 24.5 | 23.2 | 25.8 |
| **D** | 25.0 | 25.8 | 25.0 | 25.6 | 26.1 | 24.5 | 25.8 | 25.5 | 25.7 | 25.2 | 25.7 | 25.4 |
| **E** | 24.0 | 25.5 | 25.6 | 24.9 | 23.7 | 23.9 | 26.4 | 25.4 | 24.5 | 26.2 | 24.8 | 25.7 |
| **F** | 27.6 | 27.0 | 29.1 | 26.2 | 27.0 | 25.3 | 25.7 | 26.2 | 26.2 | 27.4 | 26.9 | 26.4 |

***Fig. S5:*** *Heat map of P50 (in mmHg) distribution in one exemplary ODC experiment. Gas flow is indicated by arrows.*
